# A rational design of multimodal asymmetric nanoshells as efficient tunable absorbers within the biological optical window

**DOI:** 10.1038/s41598-021-94409-9

**Published:** 2021-07-23

**Authors:** Somayeh Souri, Naby Hadilou, H. A. Navid, Rasoul Sadighi Bonabi, Abbas Anvari

**Affiliations:** 1 Department of Physics, Sharif University of Technology, Tehran, Iran; 2 Department of Laser and Optical Engineering, University of Bonab, Bonab, Iran

**Keywords:** Nanoparticles, Physical chemistry

## Abstract

In this work, the optical properties of asymmetric nanoshells with different geometries are comprehensively investigated in the quasi-static regime by applying the dipolar model and effective medium theory. The plasmonic behaviors of these nanostructures are explained by the plasmon hybridization model. Asymmetric hybrid nanoshells, composed of off-center core or nanorod core surrounded by a spherical metallic shell layer possess highly geometrically tunable optical resonances in the near-infrared regime. The plasmon modes of this nanostructures arise from the hybridization of the cavity and solid plasmon modes at the inner and outer surfaces of the shell. The results reveal that the symmetry breaking drastically affects the strength of hybridization between plasmon modes, which ultimately affects the absorption spectrum by altering the number of resonance modes, their wavelengths and absorption efficiencies. Therefore, offsetting the spherical core as well as changing the internal geometry of the nanoparticle to nanorod not only shift the resonance frequencies but can also strongly modify the relative magnitudes of the absorption efficiencies. Furthermore, higher order multipolar plasmon modes can appear in the spectrum of asymmetric nanoshell, especially in nanoegg configuration. The results also indicate that the strength of hybridization strongly depends on the metal of shell, material of core and the filling factor. Using Au-Ag alloy as a material of the shell can provide red-shifted narrow resonance peak in the near-infrared regime by combining the specific features of gold and silver. Moreover, inserting a high permittivity core in a nanoshell corresponds to a red-shift, while a core with small dielectric constant results in a blue-shift of spectrum. We envision that this research offers a novel perspective and provides a practical guideline in the fabrication of efficient tunable absorbers in the nanoscale regime.

## Introduction

Currently, there is a great interest in the development of plasmonic nanoparticles (NPs) due to their fascinating optical properties, which arise from their localized surface plasmon resonances (LSPR), in a variety of fields ranging from physics to medicine^1–5^. LSPR represents the collective oscillation of conduction electrons at the interface between a metal NP and its dielectric surrounding excited by an incident electromagnetic field. These resonances can be easily observed as pronounced peaks in the optical spectrum occur at specific frequencies and can be tuned by changing the NP’s size, geometry, material and the surrounding medium. Depending on the NP size, scattering or absorption of incident light would be the main process. The large NPs scatter the electromagnetic wave, while the dominant process for NPs less than $\sim 50$ nm in radius is absorption. Therefore, small NPs that have the ability to absorb light and convert it into local heat act as miniature heat sources^6^ and they are proper candidates for applications such as photothermal therapy (PTT), where the incident light is absorbed within the biological windows (700–1400 nm)^7,8^. In this technique, although the local temperature of tumor tissue reaches 42–47 $^{\circ }$C, the damage of healthy tissue is minimal due to their low light absorption in these spectral windows^9^. In designing efficient nanostructure for PTT applications, one must consider both tuning the plasmon energies to the wavelength of the biological window and optimizing the NP for the largest possible light absorption cross-section.

To achieve new advances, more complex geometries which provide a high degree of tunability made by combining different materials and shapes are desired. Among numerous candidates, the most widely-used nanoshells composed of a spherical core surrounded by a concentric metallic shell (i.e. core–shells NPs) show much wider spectral tunability with energies being controlled by the interaction between the individual plasmon mode supported by the inner and outer surfaces (i.e. the cavity and solid plasmon modes)^10–12^. These bi-functional NPs with an extra degree of freedom in LSPR provide high stability, low reactivity and surface modification by combining different chemical and physical properties of the core and shell parts^13–16^.

Compare to symmetric nanoshells, asymmetric ones can also provide further tunability due to a drastic change in the coupling of solid and cavity plasmon modes. When the symmetry of NP is broken, the selection rules are relaxed, so that the interactions between both different and same angular momentum numbers are allowed^17–19^. As a result, the higher order plasmon modes are excited and become visible in the optical spectrum of NP^20,21^. One can break the symmetry is giving the core an offset. For such “nanoeggs”, symmetry breaking is obtained by a displacement of the dielectric core with respect to the metallic shell^22,23^. The position of LSPRs as well as the number of higher order multipolar plasmon modes depend quite sensitively on core offset, so varying this parameter provides the tuning mechanism for this nanostructure.

Another highly anisotropic tunable nanostructure is the nanorod^24^, which can be characterized by its aspect ratio (AR), defined as the ratio between its major to minor radii. The electrons of nanorod can oscillate along the length and width of NP, hence, the optical spectrum of nanorod exhibits two distinct LSPRs corresponding to each individual electrons oscillation. Since the position of plasmon resonances is highly sensitive to the aspect ratio of the nanorod, high tunability of LSPR can be obtained by changing AR^24–26^. Besides nanoegg as a conventional tunable asymmetric NP capable of supporting multiple LSPRs, a new hybrid nanostructure that combines the optical properties of both nanoshell and nanorod, provides far greater structural tunability than either a nanorod or a nanoshell. Despite the intensity of efforts and publications, to the best of our knowledge, quantitative analysis of the asymmetric NPs, which simultaneously consider the effects of all important parameters, remains challenging.

In this work, the optical properties of asymmetric nanoshells are comprehensively investigated by applying the dipolar model and effective medium theory. In addition, the hybridization method is employed to analyze related features to the absorption spectrum. Here, the effect of symmetry breaking on the optical response of nanoshell is explored from two points of view; the former is giving the core an offset, while the latter is induced by inserting a nanorod as a core of the nanoshell. The results indicate that LSPR frequency is significantly influenced by symmetry-breaking and it can be tuned either by varying nanorod aspect ratio or core offset. Moreover, to achieve an optimum absorption spectrum, three different metals (gold, silver and their alloy) are considered as a material of the shell. Eventually, the effect of other critical factors including core aspect ratio and orientation of nanorod, core offset of nanoegg, dielectric function of the core, and filling factor are thoroughly analyzed. The rest of the paper is organized as follows: in “Model and method”, the employed theoretical method to calculate the absorption spectrum of NP is presented and important parameters are determined. Then, the effect of all important parameters is discussed in “Result and discussion”. Finally, this study is concluded in “Conclusion”.

## Model and method

A key feature that characterizes the optical spectrum of hybrid nanostructures is the interactions between different plasmon modes at different surfaces of plasmonic metal. Even the simplest possible symmetry breaking can alter these interactions and give rise to modified, and altogether new, plasmonic features. Here, two types of symmetry breaking in nanoshells are considered. One is the core offsetting which means that the spherical core is displaced and the nanoshell has a non-concentric core; the other is changing core geometry which is generated by replacing the spherical core with a nanorod. It is worth mentioning that these nanostructures are proposed based on the recent nanofabrication techniques^17,27–33^.

**Figure 1:**
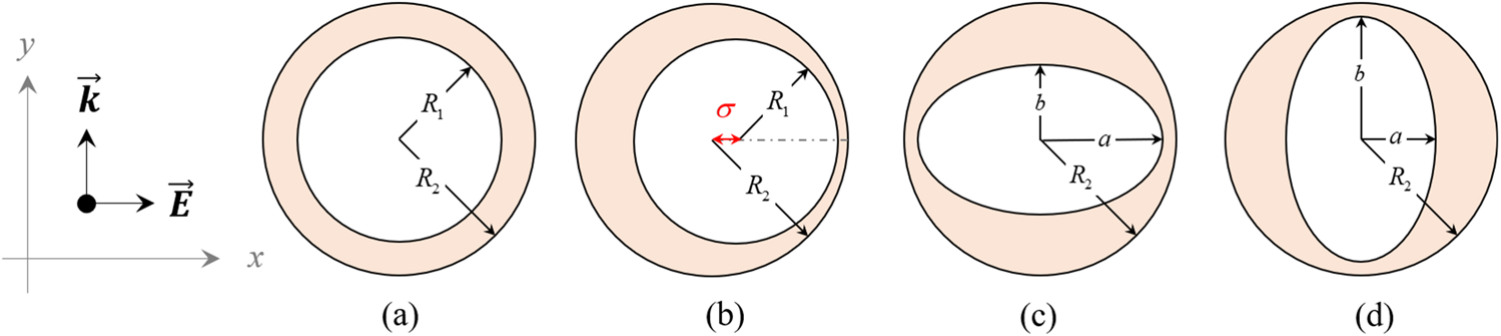
Schematic diagram of asymmetric nanoshells including (**a**) core–shell; CS, (**b**) nanoegg; NE, (**c**) prolate core in spherical shell; PS and (**d**) oblate core in spherical shell; OS.

The discussion initiates with the modal properties of the tunable asymmetric nanostructures capable of supporting multiple LSPRs. It is common to consider a nanorod as a spheroid in simulations. The spheroid is an ellipsoid with two equal axes that can look like either a stretched (prolate) or a flattened (oblate) sphere. Since the polarizability of nanorod sensitively depends on the orientation of nanorod with respect to the polarization of incident light, two distinct geometries are considered for this structure. The nanorod is treated as a prolate and oblate, when the polarization of light is parallel with its length or width, respectively. Figure 1 shows the geometry of asymmetric nanoshells consisting of a dielectric core $(\varepsilon _c)$ and metallic shell $(\varepsilon (\omega ))$ embedded in the host medium $(\varepsilon _m)$. For comparison, the schematic of symmetric core–shell nanostructure is also shown in this figure (Fig. 1a). As one can see, the first asymmetric nanoshell is a nanoegg (NE) in which the inner core $(R_1)$ is moved away from the center $(\sigma )$ but does not touch the shell $(R_2)$ (Fig. 1b). For the second asymmetric nanoshell, the hybrid NP can be achieved by inserting a dielectric core with the shape of either prolate (Fig. 1c) or oblate (Fig. 1d) in the spherical shell. The geometry of these nanoshells which called PS and OS, respectively, is defined by three parameters, minor (*a*) and major (*b*) radii of spheroid and radius of the outer spherical shell $(R_2)$. Here, it is assumed that the incident light propagates along the y-axis and is linearly polarized in the x-direction.

In the first instance, the optical response of metallic NP depends on its characteristic size *d*. If $d<< \lambda$, where $\lambda$ is the wavelength of incident light in the surrounding medium, the optical properties of NP can be discussed by the dipolar model in the quasi-static regime, where NP shows mainly a dipolar-like response. In the dipolar model, when a NP is illuminated, the external field induces a dipole moment inside the NP which is proportional to the field $\mathbf {p} = \alpha \mathbf {E}_0$, where $\mathbf {E}_0$ is the external electric field amplitude experienced by the particle and $\alpha$ is the effective polarizability of NP. Once the polarizability of the target structure is known, the scattering, absorption and extinction efficiencies can be calculated in the quasi-static limit^34^.

1 $$\begin{aligned} Q_{sca}= & {} \frac{k ^ 4}{6 \pi } |\alpha (\omega )|^2 \end{aligned}$$

2 $$\begin{aligned} Q_{abs}= & {} k \text {Im} (\alpha (\omega )) \end{aligned}$$

3 $$\begin{aligned} Q_{ext}= & {} Q_{sca} + Q_{abs} \end{aligned}$$

where $k = \frac{2 \pi }{\lambda }\sqrt{\varepsilon _m}$ is the wavenumber of light in the medium.

### Optical modeling of eccentric nanoshell

To obtain the static dipole polarizability of nanoegg $(\alpha _{NE})$, the Laplace equation was solved in two spherical coordinates by applying appropriate boundary conditions at the interfaces of core–shell and shell–environment^23,35^:

4 $$\begin{aligned} \alpha _{NE} = 4 \pi R_2^3 \frac{9\varepsilon (\omega )\varepsilon _m K_{11}(\varepsilon _c-\varepsilon (\omega ))k_2 +( \varepsilon (\omega ) -\varepsilon _m)(k_1k_2-k_3)}{(\varepsilon (\omega )+2\varepsilon _m)(k_1k_2-k_3)} \end{aligned}$$

where,

5 $$\begin{aligned} k_0&=\,  {} (\varepsilon _c - \varepsilon (\omega ))(\varepsilon (\omega )-\varepsilon _m) \end{aligned}$$

6 $$\begin{aligned} k_1&=  \,{} 2K_{11}k_0+M_{11}(\varepsilon _c+2\varepsilon (\omega ))(2\varepsilon _m+\varepsilon (\omega )) \end{aligned}$$

7 $$\begin{aligned} k_2&=\,  {} 6K_{22}k_0+M_{22}(2\varepsilon _c+3\varepsilon (\omega ))(3\varepsilon _m+2\varepsilon (\omega )) \end{aligned}$$

8 $$\begin{aligned} k_3&=  \,{} 3K_{12}M_{21}k_0(\varepsilon (\omega )+2\varepsilon _m)(3\varepsilon (\omega )+2\varepsilon _c) \end{aligned}$$

here, $K_{11} = \left( \frac{R_1}{R_2} \right)$ and $M_{11} = \left( \frac{R_2}{R_1}\right) ^2$ are coupling constants of solid and cavity dipole sphere plasmons, respectively, $K_{22} = \left( \frac{R_1}{R_2}\right) ^2$ and $M_{22} = \left( \frac{R_2}{R_1}\right) ^3$ are coupling constants of solid and cavity quadrupole sphere plasmons, respectively, and $K_{12} = 2\left( \frac{R_1}{R_2^2}\right) \sigma$ and $M_{21} = -2\left( \frac{R_2^2}{R_1^3}\right) \sigma$ are the dipole–quadrupole and quadrupole–dipole coupling constants of solid and cavity sphere plasmons, respectively. The polarizability of core–shell NP $(\alpha _{CS})$ can be obtained by setting $\sigma = 0$ in Eq. (4).

### Optical modeling of nanoshell with nanorod core

To find the static polarizability of spheroidal core in the nanoshell, the effective medium theory (EMT) can be employed. According to this analytical method, the hybrid nanostructure in a homogeneous medium is replaced by a single nanoparticle with an effective dielectric function. Using this theory, first the effective dielectric function for the entire nanostructure is derived, then a relation between the polarizability and the dielectric function through the Clausius–Mossotti relation is obtained^36,37^

9 $$\begin{aligned} \varepsilon _{i,eff} = \varepsilon (\omega )\left[ 1+\frac{f(\varepsilon _c-\varepsilon (\omega ))}{\varepsilon (\omega )+(\varepsilon _c - \varepsilon (\omega ))(L_i^c-fL_i^{sh})}\right] \end{aligned}$$

here, the core with the depolarization factor $L_i^c$ and volume $V_c$ has been considered as a spheroidal inclusion that occupies a volume fraction $f = V_c/V_{sh}$ of a spherical metallic NP with the depolarization factor $L_i^{sh}$ and volume $V_{sh}$ as a host medium $(i =x,y,z)$.

The important parameters in the geometry of an ellipsoid are its depolarization factors. The three depolarization factors for any ellipsoid satisfy $L_x + L_y + L_z = 0$. A sphere has three equal depolarization factors of 1/3. The other two special cases are prolate spheroids and oblate spheroids. For an oblate, the two major axes $(a_x < a_y = a_z)$ are equal, while for prolate, the two minor axes are of the same size $(a_x > a_y = a_z)$. The geometrical factors of oblate and prolate along their major axis are defined by Eqs. (10) and (11), respectively^34,38^.

10 $$\begin{aligned} L_{\text {major,oblate}} = \frac{1+e^2}{e^2}\left( 1-\frac{1}{e}tan^{-1}e\right) \end{aligned}$$

where the eccentricity is $e = \sqrt{1 - (b/a)^2}$

11 $$\begin{aligned} L_{\text {major,prolate}} = \frac{1-e2}{e^2}\left( \frac{1}{2e}ln\frac{1+e}{1-e}-1\right) \end{aligned}$$

$e = \sqrt{(b/a)^2 - 1}$. The depolarization factor along their minor axes is $L_{\text {minor}} = (1 - L_{\text {major}})/2$.

Once the effective dielectric function of NP is known, the dipole $(\alpha _d)$ and quadrupole $(\alpha _q)$ polarizabilities can be calculated in the quasi-static regime^39^:

12 $$\begin{aligned} \begin{aligned} \alpha _d&=\frac{\varepsilon _{eff} - \varepsilon _m}{\varepsilon _{eff} +2 \varepsilon _m} \\ \alpha _q&=\frac{\varepsilon _{eff} - \varepsilon _m}{\varepsilon _{eff} +3/2 \varepsilon _m} \end{aligned} \end{aligned}$$

The extinction and scattering efficiencies are given by Eqs. (13) and (14), respectively.

13 $$\begin{aligned} Q_{ext}= & {} 4x\text {Im}\left\{ \alpha _d + \frac{x^2}{12}\alpha _q+\frac{x^2}{30}(\varepsilon _{eff}-1)\right\} \end{aligned}$$

14 $$ Q_{{sca}}  = \frac{8}{3}x^{4} \left\{ {\left| {\alpha _{d} } \right|^{2} {\text{ + }}\frac{{x^{4} }}{{240}}\left| {\alpha _{q} } \right|^{2}  + \frac{{x^{4} }}{{900}}\left| {\varepsilon _{{eff}}  - 1} \right|^{2} } \right\} $$

where $x= k R_2$. Note that absorption efficiency is calculated as $Q_{abs}=Q_{ext} - Q_{sca}$.

From Eqs. (4), (9) and (12), it is apparent that the polarizability of NP contains various parameters that have their own significance. Out of these parameters, the dielectric function is one of the most important parameters to understand the optical properties of metallic nanoshell. Drude–Lorentz model gives the size dependent dielectric function of the NP as^40–43^:

15 $$\begin{aligned} \varepsilon (\omega )=\varepsilon _{\infty }+\frac{\omega _p^2}{\omega ^2+i\omega \gamma _{bulk}}-\frac{\omega _p^2}{\omega ^2+i\omega (\gamma _{bulk}+\gamma )} \end{aligned}$$

$\varepsilon _{\infty }$ is the dielectric constant of bulk metal, $\omega _p$ is the plasma frequency, $\omega$ is the photon energy and $\gamma _{bulk}$ is the electron collision damping in the metal. For small nanoshells, i.e. smaller than 50 nm, the dielectric function has to be modified due to the size dependence of electron mean free path. The finite size effect on the dielectric function of NP is considered by $\gamma = Av_f/L_{eff}$, where *A* is a dimensionless parameter for matching theoretical and empirical data, $v_F$ is the Fermi velocity and $L_{eff}$ is the effective mean free path of electron which can be calculated as^44^:

16 $$\begin{aligned} L_{eff} = \frac{4V}{S_{in}+S_{out}} \end{aligned}$$

where, V is the volume of the nanoshell. $S_{in}$ and $S_{out}$ represent the internal and external surfaces of the shell, respectively.

Since, gold and silver in NP form exhibit the most interesting selective absorption in the visible and near-infrared regime, here, the optical properties of nanoshells in which the metallic shells are one of these two materials or their combination in the form of alloy, is investigated. The related data to the dielectric function of gold and silver are presented in Table 1^45^. The dielectric constant of the core and host medium can be mentioned as other important parameters. Here, the core is made of silica and nanoshell is embedded in the aqueous environment. In the wavelength range of 300–1400 nm, the refractive index of ${\text {SiO}}_2$ and water have been measured as 1.47 and 1.33, respectively.

**Table 1:** Key parameters value of gold and silver.

Metal	$\varepsilon _{\infty }\;{\text {(eV)}}$	$\omega _p\;{\text {(eV)}}$	$\gamma _{bulk}\;{\text {(eV)}}$	$v_F\;{\text {(m/s)}}$
Gold	9.8	9	0.066	$1.4 \times 10^6$
Silver	3.7	8.9	0.018	$1.39\times 10^6$

## Result and discussion

### Geometry

**Figure 2:**
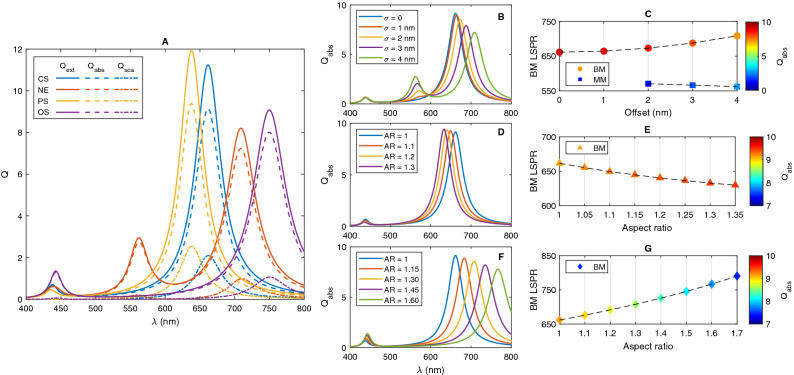
(**A**) Optical response of ${\text {SiO}}_2 @ {\text {Au}}$ nanoshells with different core geometries. Absorption spectrum of (**B**) NE with different core offset $(\sigma )$, (**C**) effect of varying core offset on the LSPR wavelength and its absorption efficiency of NE, (**D**) absorption spectrum of PS with different aspect ratio (AR) and (**E**) effect of AR on LSPR and absorption efficiency of PS. (**F**) Absorption spectrum of OS with different AR and (**G**) effect of aspect ratio on LSPR and absorption efficiency of OS. The total radii of NPs are fixed at 25 nm and dimension of each spheroidal core was chosen so that the volume of core is equal to that for a 20 nm radius sphere.

Here, the optical response of nanoshell with different geometries including core-shell (CS), nanoegg (NE), core with prolate shape (PS) and core with oblate shape (OS), are investigated. For a reasonable comparison between different geometries of NP, the radius of the shell and filling factor (*f* is the filling factor, defined as the ratio of the core to nanoparticle volumes) are identical for all structures. A spherical nanoshell with outer radius of 25 nm and filling factor of $f=0.512$ is considered. For a nanoshell with a spherical core, the radius of the core is 20 nm. In the case of nanoegg, the offset is considered to be in the range of $\sigma$ = 1–4 nm. For PS and OS nanostructures, the major and minor radii are varied to achieve different aspect ratios, while the volume of the core is fixed. Figure 2A reports the optical response of nanoshells. Here, the core offset of NE is 4 nm and for PS and OS nanostructures, (*a*, *b*) are (23 nm, 15.12 nm) and (18.65 nm, 23 nm), respectively. For all structures presented in this part, the metal and dielectric of choice are gold and silica, respectively. It is found that the scattering efficiencies are smaller than the absorption ones and most part of the incident light is absorbed by these small nanoshells. Therefore, only absorption spectra are presented in the next figures.

As one can see, two distinct plasmon resonance peaks can be observed in the spectra of CS, PS and OS; while the spectrum of NE exhibits three LSPR peaks.To understand this plasmonic behavior, the plasmon hybridization of solid and cavity plasmon modes can be applied to this geometry. According to the hybridization model, metallic nanoshell supports plasmon modes at the inner and outer surfaces of the shell which can interact with each other due to the finite thickness of the shell^46–48^. This interaction results in the splitting of the plasmon resonances into two hybridized resonances; the lower energy bonding mode (BM) and the higher energy antibonding mode (ABM). The strength of hybridization between plasmon modes is controlled by the thickness of the metallic shell. The coupling between incident light and BM is strong due to a large net electric dipole moment of bonding mode,whereas there is a weak interaction between the optical field of light and ABM with an electric quadrupolar nature^49,50^.

**Figure 3:**
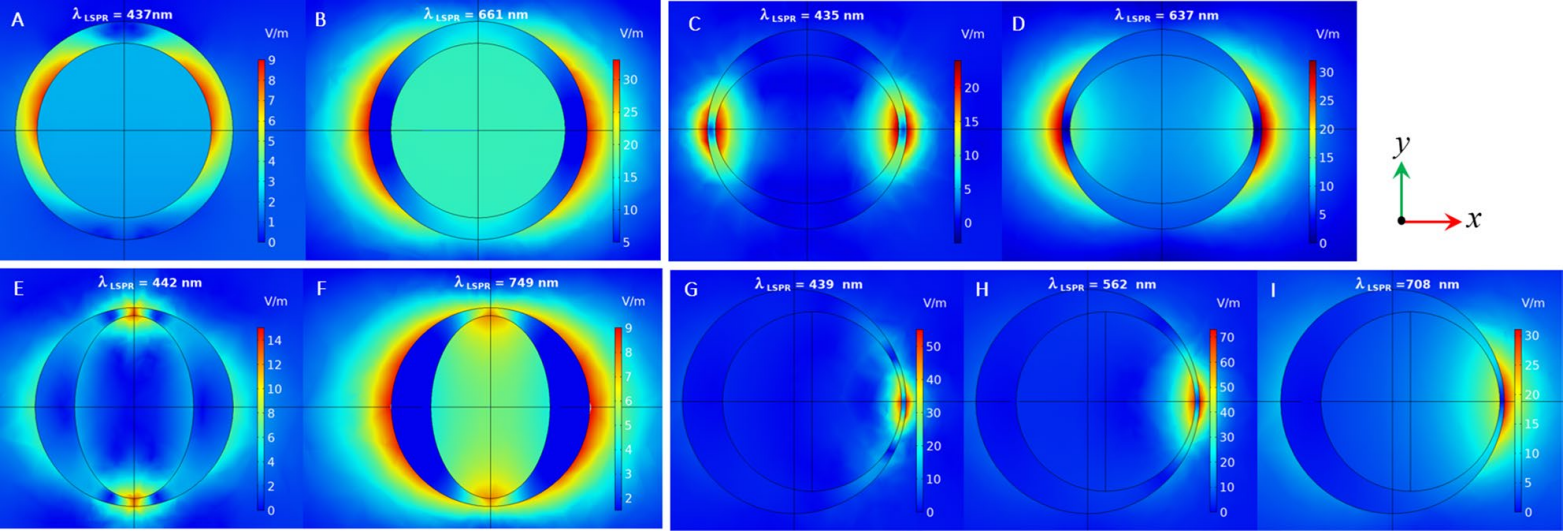
Electric field distribution of (**A**, **B**) CS, (**C**, **D**) PS and (**E**, **F**) OS at ABM and BM, respectively. (**G**, **I**) Electric field distribution of NE at ABM, MM and BM, respectively.

As it can be seen from Fig. 2A, both BM and ABM are observed in the absorption spectra of CS, PS and OS structures. The position of Low-energy plasmon modes of CS, PS and OS are 661 nm, 637 nm and 749 nm and their high-energy modes are located at 437 nm, 435 nm and 442 nm, respectively. Besides antibonding and bonding modes at 439 nm and 708 nm, another resonance mode is also observed in the spectrum of NE at 562 nm. To gain further insights into the plasmon modes, the electric field distributions near the nanoshells for all LSPR maxima are also calculated and presented in Fig. 3. It is evident that there is a high field enhancement at the outer interface of nanoshells for the bonding resonance modes, whereas the maximum electric field is strongly confined inside the nanoshell for the antibonding modes. Unlike CS, Ps and OS with almost symmetric field distribution at the outer surface, the electric field is not uniformly distributed at the outer surface of NE, instead, it is concentrated at the side where the core is close to the shell. On the other side, almost no electric field is observed. Note that the highest field enhancement can be achieved by NE at 562 nm.

**Figure 4:**
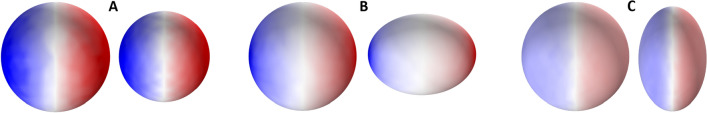
Surface charge density (Coulomb/m$^2$) plots of shell and core surfaces of (**A**) CS, (**B**) PS and (**C**) OS for bonding mode.

As the geometry of the spherical core alters to the elongated spheroids, the charge accumulation on the surface of the core, the thickness of the shell and therefore, the energy of the cavity plasmon mode which ultimately affects the hybridization, change. As a result, the position of BM and AMB peaks and their absorption efficiencies can be tuned. It is well known that the polarizability of NP determines the absorption efficiency and its strength extremely depends on the magnitude of charges on the surface of the nanoparticle^51^. This charge accumulation is influenced by the NP shape. Depends on the polarization of the incident light, charge separation on the surface of spheroidal NP is in two direction. Since the incident radiation is linearly polarized in the x-direction, the longitudinal and transverse plasmon modes are excited in the prolate and oblate, respectively. For more accurate discussion about charge accumulation, the surface charge density at the metal-dielectric interfaces of nanoshell, should be considered^52,53^. Surface charge density is calculated by applying Gauss’s law at the inner and outer surfaces of nanoshell^21^. Figure 4 shows the surface charge density on the inner and outer surfaces of nanoshells for bonding plasmon modes. Obviously, due to the light propagation, opposite charges are found on the surface along the electric field direction in which light is polarized (x-axis). The same induced charges residing on the inner and outer surfaces of the nanoshell, indicating the dipolar bonding plasmon mode. From Fig. 4, it is clear that when the spherical core deforms toward elongated spheroids, the magnitude of the effective surface charge density as well as its spatial separation are changed^54,55^. When the electric field is transverse to the long axis of the spheroid (OS—Fig. 4C), surface charges are generated on the sides of the oblate. In this case, the magnitude of induced charges and their spatial separation are decreased. Here, the effect of charge reduction is much greater, and therefore, the restoring force is reduced. This corresponds to the red-shift of the cavity plasmon mode. On the contrary, when the electric field is parallel to the long axis of the spheroid (PS—Fig. 4B), surface charges are generated on the tips of the prolate and the situation is reversed and results in a blue-shift of the cavity plasmon mode^54,56^.

It has been shown that the absorption peaks are incredibly sensitive to varying electron density distributed on the inner and outer surfaces of nanoshell. The shift of LSPR peak can be calculated by Eq. (17)^57^

17 $$\begin{aligned} {\Delta \lambda =\lambda _{sp}\frac{\Delta N}{N}\sqrt{\varepsilon _{\infty }+\frac{1-\eta }{\eta }\varepsilon _m}} \end{aligned}$$

where $\lambda _{sp}$, *N* and $\eta$ are the LSPR wavelength, the electron density and the particle shape factor, respectively. For OS structure, where coupling occurs between the dipolar mode of the shell and the transverse mode of the spheroidal core, there is a great change in the electron density. Therefore, a considerable red-shift is observed in the bonding mode. It seems that the hybridization between inner and outer plasmon modes becomes stronger which results in a larger energy gap between BM and ABM. In addition, the coupling between ABM and incident optical field is enhanced since this mode can have a net electric dipole moment. Although the resonance occurs at a higher wavelength and shifts toward the near-infrared regime, this plasmon mode is relatively more broadened. For PS, the situation is reversed and BM mode experience a minor blue-shift due to a decrease in electron density. Here, the hybridization is weak and a small splitting of the plasmon modes will occur. However, narrowing the spectrum is the main advantage of this structure.

As mentioned, another interesting outcome from Fig. 2A is the emergence of a new peak in the absorption spectrum of NE. The reason is as follows; For CS with a concentric core, plasmon hybridization only occurs between solid and cavity modes of the same angular momentum numbers. However, when the center of the core is displaced with respect to the center of the shell, i.e. nanoegg, the selection rules for the interaction of plasmon modes are strongly modified and the hybridization of solid and cavity plasmons with different angular momenta is allowed. This hybridization leads to a red-shift of both BM and ABM to the lower energy and appears new resonance due to coupling between dipole-quadrupole cavity and solid plasmon modes. Multiple LSPR modes makes NE a good candidate for applications where two or more simultaneous absorption enhancement at different wavelengths is required.

The results reported in Fig. 2A indicate that inserting an asymmetric core into a spherical nanoshell can provide more tunable LSPR by changing core offset of NE or aspect ratio $(AR = \text {Major radius}/\text {Minor Radius})$ of spheroidal core, in comparison with concentric nanoshell. The absorption spectra of NEs with different core offsets are plotted in Fig. 2B. It is clear that only BM and ABM are observed in the absorption spectrum when the core offset is small. With the increase of core offset, the interaction of dipole–quadrupole modes leads to emergence of a new LSPR mode (MM) in the spectrum. A larger offset correlates with a larger red-shift and smaller absorption efficiency in BM LSPR. The position and efficiency of ABM, on the other hand, almost remain constant. Figure 2C provides the detail of the core offset effect on LSPR wavelength and absorption efficiency of BM and MM. Figure 2D,F show the absorption spectra of PS and OS, respectively, where the aspect ratio of the core is varied by keeping the NP size and core volume fraction fixed. It has been found that BM LSPR of PS and OS experience a slight blue-shift and significant red-shift when the AR is increased, respectively. Based on the findings, by changing AR, the BM peak of OS can be considerably tuned from 661 to 790 nm and the BM peak of PS can be slightly adjusted from 661 to 620 nm. ABM mode of both structures almost remains constant. Despite the advantage of high resonance displacement, the absorption efficiency of OS NP is reduced by increasing AR. On the contrary, this parameter does not significantly affect the absorption yield of PS nanostructure. The effect of AR of PS and OS on the LSPR wavelength and its absorption efficiency is reported in Fig. 2E,G, respectively.

### Material

The absorption spectra of NPs are fully characterized by the resonance frequency, the maximum absorption cross-section and the bandwidth of the LSPR^58^. For photothermal applications, nanoparticles should possess narrow LSPR with high absorption within the biological window. Since gold has a smaller real part and greater imaginary part of the dielectric function, less polarized charges and more plasmonic damping are produced by Au NPs compared to Ag NPs^59,60^. In other words, the Au-LSPR always occurs at a higher wavelength as compared with the ones calculated for the Ag NP. However, the absorption spectrum of gold is broadened and its maximum is much less than the sharp LSPR peak of silver. In addition, Au NPs have good biocompatibility and high chemical stability, while Ag NPs show toxicity and chemical instability. Combining gold and silver as shell material demonstrates fascinating optical properties in comparison with monometallic NPs. Au–Ag alloy would merge the properties of high homogeneity and biocompatibility of gold and higher absorption cross-section of silver^61^. The use of this alloy as a nanoshell material provides two advantageous; red-shifted LSPR to NIR regime and narrow bandwidth spectrum. Moreover, further tunability can be obtained, since the percentage of each constituent can be varied.

To investigate the effect of the metallic shell material, the absorption spectra of 20 nm nanoshells (CS, NE, PS and OS) with different metallic shells (Au, Ag and alloy) embedded in the water were calculated. Note that the dielectric function of alloy $\text {Au}_x\text {Ag}_{1-x}$ can be measured using a simple linear combination of the dielectric constants^62^

18 $$\begin{aligned} \varepsilon _{\text {Alloy}}(\omega ) = x\varepsilon _{\text {Au}}(\omega )+ (1-x)\varepsilon _{\text {Ag}}(\omega ) \end{aligned}$$

where *x* denotes the Au fraction in the alloy.

**Figure 5:**
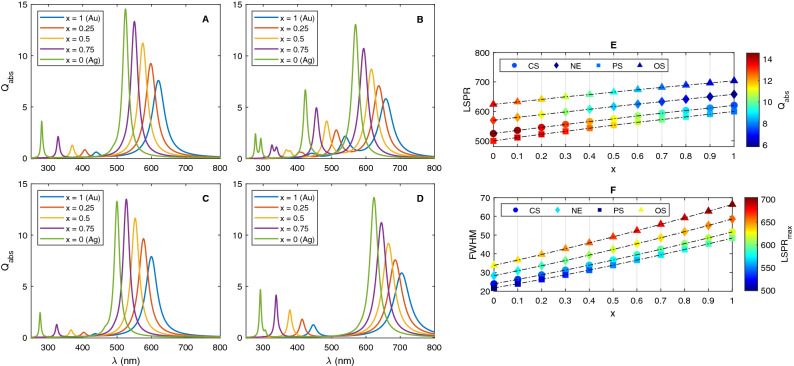
Absorption spectra of Au–Ag alloy-coated (**A**) Cs, (**B**) NE, (**C**) PS and (**D**) OS NPs with different gold fractions. Variation of (**E**) LSPR, absorption efficiency and (**F**) bandwidth of resonance peak with gold fraction in the alloy as shell material. The total radii of SiO$_2$@ Au$_x$Ag$_{1-x}$ are fixed at 20 nm and a dimension of each core have been chosen so that the volume of core is equal to that for a 15 nm radius sphere.

The results in Fig. 5 clearly demonstrate how the absorption spectra get red-shifted and absorption efficiency is reduced as Au content in the nanoshell material increases. This trend is observed for all structures (CS, NE, OS and PS). On the contrary, the structures with higher content of Ag have narrower spectra with greater absorption efficiencies. Pinchuk et al reported that the additional contribution of the interband electronic transitions should be considered in the dielectric function of noble metal^58^. Therefore, the resonance frequency and full width at half maximum (FWHM) of the LSPR can be modified as

19 $$\begin{aligned} \omega _{LSPR}= & {} \frac{\omega _p}{\sqrt{2\varepsilon _m + \chi + 1}} \end{aligned}$$

20 $$\begin{aligned} \text {FWHM}= & {} \gamma \sqrt{1 + \frac{2 \text {Im}(\chi ) \omega _p}{\gamma (1 +\text {Re}(\chi ) + 2 \varepsilon _m)}(1 + 2 \varepsilon _m)^{1/2}} \end{aligned}$$

where $\chi$ is the interband susceptibility^63^. Since $\chi _{Au} > \chi _{Ag}$, the LSPR of Au occurs at higher wavelengths. For silver, the imaginary part of susceptibility is considerably small, so that $\text {Im}(\chi ) \approx 0$ in the optical range. As a result, the square root in Eq. (20) is about unity and FWHM approximately equals damping rate. However, the plasmonic mode of the Au is broadened as compared to the one for Ag, due to the more contribution of the imaginary part of the interband transition to the bulk dielectric function of gold. It is also observed that the absorption efficiency is enhanced by increasing the amount of Ag in nanoshells due to the large absorption coefficient of silver^64^. Obviously, the amplitude of the absorption spectrum is roughly determined by absorption coefficient, $\alpha (\omega )=2\kappa (\omega ) \omega /c$ where $\kappa (\omega ) = \text {Im}(\varepsilon )/(2n)$; $\kappa$ and *n* is extinction coefficient and refractive index, respectively. The detail of the Au fraction effect on BM LSPR wavelength, FWHM and absorption efficiency is presented in Fig. 3E,F.

### Core dielectric function

**Figure 6:**
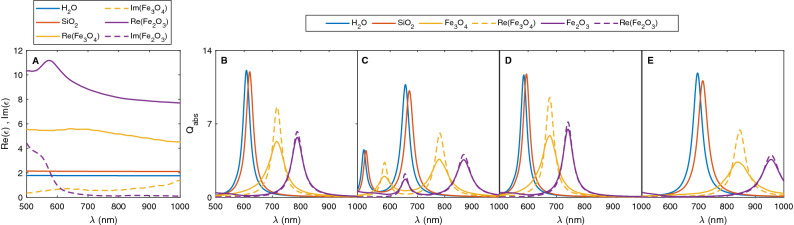
(**A**) Dielectric function of water, silica, magnetite and hematite. LSPR spectra of (**B**) CS, (**C**) NE, (**D**) PS and (**E**) OS with core materials of water, silica, magnetite and hematite. The total radius of the nanoshell is 25 nm and the core occupies 51% of the nanoshell.

It is apparent that both the LSPR peak position and intensity of absorption spectra are significantly influenced by the dielectric properties of the core materials. Using magnetic core in nanoshell offers the unique advantage of a combination of magnetic and plasmonic properties. The most commonly used magnetic NPs in biological application are iron oxides, which have several different compositions, such as magnetite $(\text {Fe}_3\text {O}_4)$ and hematite $(\text {Fe}_2\text {O}_3)$. Unlike silica, which is transparent to visible light and has a near-zero imaginary component of permittivity, iron oxide has complex permittivity in which the imaginary component accounts for the absorption of light. To exemplify the effect of core dielectric and take advantage of magnetoplasmonic nanostructures, the optical properties of nanoshells are calculated for four different core materials; hollow, which has a refractive index equal to that of the surrounding environment, silica, magnetite and hematite. Figure 6 presents the absorption spectra of nanoshells, identical to those in Fig. 2A, except that the alloy $\text {Au}_{0.5}\text {Ag}_{0.5}$ has been chosen as a shell material. Note that the dielectric constant of iron oxide varies with the wavelength of incident light in the visible-infrared range^65^. Here, only the bonding modes are analyzed because they are the most intense plasmonic modes in the optical spectra we are interested in; antibonding modes are not observable in the selected spectra (500–1000 nm).

The results reveal that a difference in the values of the core dielectric constants leads to the shift of LSPR peak due to a modification of cavity plasmon mode which results in the change of the extent of the plasmon coupling. When the core region is filled by a higher dielectric medium, the effective dielectric constant of the core will be increased and results in a greater polarization of the dielectric medium. As a consequence, the accumulated charges in the resonance zone will attenuate and therefore, the restoring force can be reduced. This corresponds to the lower resonant frequency (i.e., larger wavelength). This is why resonance peaks experience red-shifted^66,67^. Filling a lower dielectric medium in the core regions reverses the situation. In other words, a core with a high permittivity corresponds to a red-shift, whereas a core with small dielectric constant results in a blue-shift of LSPR. Compared to $\text {SiO}_2@\text {Au}_{0.5}\text {Ag}_{0.5}$, hollow nanoshell is slightly blue-shifted while, the spectra of $\text {Fe}_3\text {O}_4@\text {Au}_{0.5}\text {Ag}_{0.5}$ and $\text {Fe}_2\text {O}_3@\text {Au}_{0.5}\text {Ag}_{0.5}$ are considerably red-shifted; the larger is the real refractive index, the larger is the red-shift of the LSPR peak. It is also observed that the LSPR peak intensity of the iron oxide II and III are remarkably lower than those of the hollow-core and silica-core NPs. The dashed lines show the absorption spectra of iron oxides with only the real part of the refractive index for comparison. It is clear that the position of LSPR peaks are identical for absorbing and non-absorbing magnetoplasmonic NPs; however, the absorption of the absorbing ones is lower. The reason is that the strength of hybridization between solid and cavity plasmon modes decreases when a core material with a complex refractive index is used due to dampening the cavity plasmon modes^68^. Therefore, when the imaginary component of permittivity is not negligible, the intensity of the LSPR peak is weakened; the larger is the imaginary refractive index, the larger is intensity drop of the LSPR peak. It should be noted for $\text {Fe}_3\text {O}_4$ and $\text {Fe}_2\text {O}_3$, the imaginary component of refractive index in the optical range of (500–1000 nm) is small; hence, its effect on the position of resonance peak is negligible^69^.

### Filling factor

**Figure 7:**
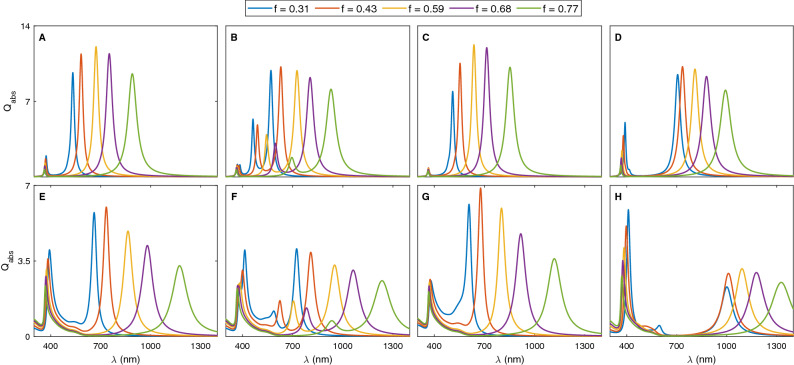
Absorption spectra of (**A**–**E**) CS, (**B**–**F**) NE, (**C**–**G**) PS and (**D**–**H**) OS configuration with different filling factors. Top panel: SiO$_2$@ Au$_{0.5}$Ag$_{0.5}$. Bottom panel: Fe$_2$O$_3$@ Au$_{0.5}$Ag$_{0.5}$. The total radii of nanoshells are fixed at 25 nm. For Ps and OS, the major radii are 24 nm.

The impact of the filling factor (*f*) on the tunability of the resonance peak as well as the absorption efficiency is investigated by changing the core dimension while the total size of the nanoshell remains constant. For a complete comparison, the absorption spectra of metal-coated silica and hematite NPs embedded in water for CS, NE, PS and OS configurations are calculated and shown in Fig. 7. Here, the metal of choice is an alloy containing 50% gold. The nanoshells radii are set with a fixed value of 25 nm, whereas the values of *f* are increased from 0.31 to 0.77.

For all configurations, the results clearly indicate that the resonance peak at a longer wavelength shows a considerable red-shift, whereas the resonance peak at a short wavelength exhibits a slight blue-shift when the ratio between the core and NP volumes (*f*) is increased. Since these two modes exhibit opposite trends in the peak position displacement, it is concluded that well-separated LSPRs can be obtained at higher *f*. This trend is observed in the spectra of both $\text {SiO}_2 @ \text {Alloy}$ and $\text {Fe}_2\text {O}_3 @ \text {Alloy}$. As expected, a larger red-shift is observed in the absorption spectrum of magnetic NP due to a larger real part of dielectric permittivity. At the same time, it can be seen that higher order plasmon modes are excited and additional LSPRs appear in the spectrum of NE and OS for small *f* due to a relatively strong symmetry breaking in these structures. Moreover, the absorption efficiency of BM mode initially increases, reaches its maximum and then decreases. In addition, for MM resonance peak in NE configuration, the considerable red-shift alongside absorption reduction is observed in the spectrum by increasing the value of *f*.

Another effect seen in Fig. 7 is that the absorption intensity of the hybridized mode at shorter wavelength is several times enhanced, for $\text {Fe}_2\text {O}_3 @ \text {Au}_{0.5}\text {Ag}_{0.5}$. In other words, the antibonding mode becomes visible in the absorption spectrum. As mentioned before, the properties of nanohybrid structures could be described in terms of plasmon hybridization, where the cavity plasmon modes associated with the inner surface of the shell interact with the solid plasmon modes at the outer surface of the shell. Apart from the geometric parameters, the energy of the cavity mode is determined by the dielectric permittivity of the core and shell, while the energy of the solid mode depends on the permittivity of the shell and surrounding medium^49^. Therefore, any variation in $\varepsilon _c$ leads to an adjustment of cavity mode’s energy and a change in the absorption spectrum. For the case of a nanoshell with a low dielectric permittivity core, i.e. silica, the cavity plasmon mode is at higher energy than the solid plasmon mode. As a result, the low and high energy hybridized modes are bright and dark plasmon, respectively and the absorption spectrum is dominated entirely by the low energy mode. Contrary, for the case of a high permittivity core material; i.e. hematite, the energy of the cavity plasmon mode is lower than the energy of solid mode. In this regime, although the bonding mode is present in the absorption spectrum, the higher energy antibonding mode will have the largest absorption efficiency^68^. It should be pointed out this absorption enhancement is much stronger in OS NPs, so that the absorption of ABM is several times larger than those for BM. Recall that the difference in the absorption amplitude would be attributed to the difference in the charge separation^63^. Except for PS nanostructure with a slight change in the absorption efficiency of ABM mode, $\text {Q}_\text {abs}$ gradually decreases as the filling factor increases.

**Figure 8:**
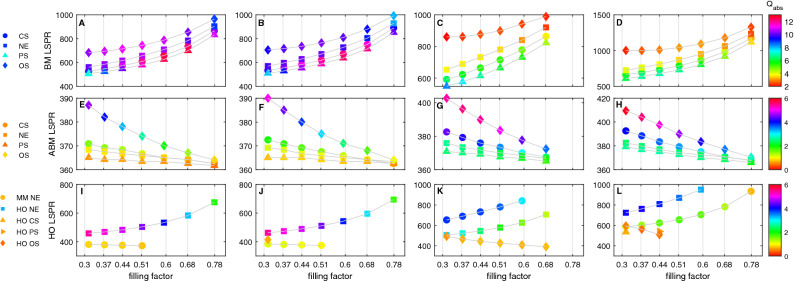
Effect of varying the filling factor on the (top panel) BM, (middle panel) ABM and (bottom panel) higher order; HO; LSPR wavelength and its absorption efficiency of (**A**, **E**, **I**) H$_2$O@ Au$_{0.5}$Ag$_{0.5}$, (**B**, **F**, **J**) SiO$_2$@ Au$_{0.5}$Ag$_{0.5}$, (**C**, **G**, **K**) Fe$_3$O$_4$@ Au$_{0.5}$Ag$_{0.5}$ and (**D**, **H**, **L**) Fe$_2$O$_3$@ Au$_{0.5}$Ag$_{0.5}$.

Similarly, the effect of the filling factor on the optical properties of NPs with core materials of hollow and magnetite is also analyzed. Figure 8 provides the detail of the filling factor effect on the absorption spectrum of NPs with different geometries; CS, NE, PS and OS. The results signify that the filling factor is an important parameter for tuning the resonance in the wavelength range from 300 to 1400 nm.

## Conclusion

In this paper, a detailed theoretical study of the optical response of asymmetric nanoshells with different geometries including core–shell, nanoegg and nanorod core in a spherical shell (i.e., PS and OS) in the quasi-static regime by applying the dipolar model and effective medium theory has been presented. The plasmon hybridization model is employed to explain the plasmonic behavior of these nanostructures. According to hybridization theory, the interaction of solid and cavity plasmon modes at the inner and outer surfaces of nanoshell results in the splitting of the plasmon resonances into a lower energy bonding mode (BM) and a higher energy antibonding mode (ABM). Additionally, the higher order plasmon modes can be excited and appear in the absorption spectrum due to the relaxation of selection rules of plasmon interactions. The strength of this coupling is remarkably altered either by changing the geometry of the core to prolate or oblate or by offsetting the spherical core. In comparison with CS, the considerable red-shift alongside absorption reduction is observed in the BM mode of PS and OS, while the shift of ABM modes is slight. For the case of nanorod as the core, the results indicate that the coupling between plasmon modes of OS is stronger and the energy gap between BM and ABM is larger. The absorption spectrum of OS shifts toward the higher wavelengths at the near-infrared regime. Moreover, the ABM mode of OS is also enhanced due to its large electric dipole moment. On the other side, although the red-shift of PS is not as large as OS, LSPR is much narrower than the one for OS. For NE, besides BM and ABM, another resonance mode (MM) emerges in the spectrum as a result of coupling between the cavity and plasmon modes with different angular momenta. In addition, the bonding mode of NE is also red-shifted and its absorption is decreased, compared to CS. Furthermore, the absorption spectrum of asymmetric nanoshell can be further tuned over the wider spectral region either by changing the aspect ratio of nanorod or core offset of nanoegg. Based on findings, a larger aspect ratio correlates with a larger shift in the spectrum. Similarly, a larger core offset leads to a larger red-shift of the spectrum.

It is also found that the optical response of nanoshell significantly depends on the material of the shell. In contrary with the silver-coated nanoshell which provides narrow LSPR at shorter wavelengths with high absorption efficiency, the more broaden LSPR with less absorption of gold-coated NP occurs at longer wavelengths. Combining gold and silver as the shell material is a good method to merge the advantages of both gold and silver in single NP. In addition, the absorption spectrum is tuned over the wide spectral range by changing the percentage of gold or silver in the alloy. When Au content in the alloy increases, the spectrum gets red-shifted and absorption efficiency is reduced. The same trend is observed in the spectra of all geometries; CS, NE, PS and OS.

One of the effective parameters to modify the hybridization is the dielectric function of the core since it determines the cavity plasmon mode energy. Therefore, changing the material of the core leads to a change in the optical response of nanoshell. Inserting a high permittivity core in the nanoshell corresponds to a red-shift, while a core with small dielectric constant results in a blue-shift of bonding mode. On the other side, using a magnetic core (magnetite and hematite) with a complex dielectric function in the metallic nanoshell can unite the plasmonic and magnetic properties in a single NP. The larger red-shift is observed in the spectra of magnetite and hematite due to their large real refractive indices. At the same time, the absorption intensities of LSPR of magnetic NPs are remarkably lower than one of the silica-core nanoshell due to the decrease in the strength of hybridization between solid and cavity plasmon modes as a result of dampening the cavity plasmon modes by their imaginary part of dielectric functions.

Finally, it is observed that there is a significant red-shift in the resonance peaks at longer wavelengths as the filling factor increases, while the resonance peaks at short wavelengths exhibit a slight blue-shift. This trend is observed in the absorption spectra of all reported NPs. These findings open a new route to optimize the synthesis of hybrid nanostructures in various applications as far as absorption is concerned.
